# Vivaldi Antenna Arrays Feed by Frequency-Independent Phase Shifter for High Directivity and Gain Used in Microwave Sensing and Communication Applications

**DOI:** 10.3390/s21186091

**Published:** 2021-09-11

**Authors:** Jiwan Ghimire, Feyisa Debo Diba, Ji-Hoon Kim, Dong-You Choi

**Affiliations:** 1 Department of Information and Communication Engineering, Chosun University, 375 Susuk-dong, Dong-gu, Gwangju 501-759, Korea; ghimire@chosun.kr (J.G.); feyisa@chosun.ac.kr (F.D.D.); 2 Department of Electronics and Communication Engineering, Adama Science and Technology University, Adama 1888, Ethiopia; 3 Department of Mechanical Engineering, Chosun University, 375 Susuk-dong, Dong-gu, Gwangju 501-759, Korea; kjh@chosun.ac.kr

**Keywords:** ultra-wideband antenna, Vivaldi antenna array, power dividers, ground penetration radar

## Abstract

This paper describes a novel feed system for compact, wideband, high gain six-slot Vivaldi antenna arrays on a single substrate layer using a unique combination of power splitters based on binary T-junction power splitter topology, frequency-independent phase shifter, and a T-branch. The proposed antenna system consists of six Vivaldi antennas, three on the left, and three on the right arm. Each arm connects with T-junction power divider splitter topology, given that the right arm is linked through a frequency-independent phase shifter. Phase shifters ensure that the beam is symmetrical without splitting in a radiating plane so that highly directive radiation patterns occur. The optimal return losses (S-parameters) are well enriched by reforming Vivaldi’s feeding arms and optimizing Vivaldi slots and feeds. A novel feature of our design is that the antenna exhibits the arrangements of a T-junction power splitter with an out-of-phase feeding mechanism in one of the arms, followed by a T-branching feeding to even arrays of proper Vivaldi antenna arrangement contributing high realized gain and front-to-back ratio up to 14.12 dBi and 23.23 dB respectively applicable for not only ultra-wideband (UWB) application, also for sensing and position detecting. The high directivity over the entire UWB frequency band in both higher and lower frequency ranges ensures that the antenna can be used in microwave through-wall imaging along with resolution imaging for ground penetration radar (GPR) applications. The fabricated antenna parameters are in close agreement with the simulated and measured results and are deployed for the detection of targets inside the voids of the concrete brick.

## 1. Introduction

In a modern context, wireless devices have seen immense growth in the field of radar, radio astronomy, vehicular, high-speed communication, surveillance, and UWB imaging systems, making communication tools and techniques easily accessible and fast [1,2,3]. Due to certain benefits over conventional antennas such as their low profile, compactness, cheap costs, planar configuration, suitability for arrays, and ease of integration [4,5], a microstrip antenna has been considered as a potential alternative for detecting landmines [6], through-wall imaging [7], biomedical imaging [8], breathing and heart rate detection [9,10], motion and gesture detection [11], and a two-dimensional (2D) imaging of concrete block [12]. Most of the UWB antennas existing in the literature had to overcome high propagation loss within microwave bands confined by a specific transmit spectrum. For microwave imaging of the buried targets, voids, and hidden cracks in concrete-based construction material and composite structure, the antenna with a wide frequency band, stable unidirectional end-fire radiation character, and a high directive gain is preferable to get desired penetration depth, especially within high lossy material. The traditional destructive methods for checking are time-consuming, dangerous, and expensive, so the use of non-destructive testing (NDT) is preferred for subsurface monitoring where a high depth and range resolution tracking antenna is required. Different antenna designs are implemented for UWB GPR systems such as dipole [13], bow-tie antenna [14,15], the spiral antenna [16], tapered slot antenna [17,18], and TEM horn antenna [19,20] depending upon whether the application demand requires high penetration depth with low resolution or vice-versa. The GPR antennas have either low gain or are bandwidth limited, can exhibit late-time ringing while transmitting short-time pulse, and are bulky [21]. The bandwidth of the antenna plays an important role in selecting the application requirement for a high depth penetration with low resolution or vice-versa. The choice between penetration depth and resolution is determined by the antenna transmission frequency, so a high bandwidth antenna is required for the desired frequency selection. Ground-coupled and air-coupled antennas are commonly employed for GPR assessment, however, for detection of layer interference such as voids and targets, air-coupled antennas are preferable [22]. Because airborne GPR antennas still have severe disputes with broad bandwidth, narrow beam width, light weight, and high gain, these issues can be addressed by deploying a planner antenna like Vivaldi [23]. The Vivaldi antenna and its array could be suitable for microwave communication for their wide bandwidth, easy fabrication, high efficiencies and directivity, simple structure, consistency in E-H plane, and lightweight characteristics [24]. However, the conventional Vivaldi antenna in an array undergoes some weaknesses, like slanting beam and changeable in directivity and gain at higher frequency [25,26]. To overcome these problems, several methods have been proposed for achieving higher gain by using methods like employing dielectric lens [26], metamaterial lens [27,28], parasitic elliptical patch [29], and electromagnetic bandgap (EBG) [30]. Other methods include use of profiled dielectric directors [31,32], modification of radiating arm slots [33], array structure [34,35], negative index material (NIM) [36], zero-index material (ZIM) [37] and dielectric slab [38]. However, developing efficient gain enhancement with different existing techniques in an area of limited size within a planner antenna system is still a costly, complicated, and difficult task. Array structures are generally used to achieve high gain with inevitable problems of the side lobes and mutual coupling in array design. The solutions to solve the ambiguity between the side lobes and the mutual coupling of the corresponding radiating elements arise while tuning the antenna’s performance. High coupling causes significant degradation of antenna gain and bandwidth while increasing the side lobes can cause a decrease in antenna radiation performance. The wideband performance can be achieved if the array elements are electrically connected to adjacent elements [39], whereas by increasing the aperture size of the radiating patch, the lower frequency response will be satisfactory along with the improvement in the radiation characteristics [40,41]. The performance of an antenna, particularly in Vivaldi arrays, is determined by its feeding network. The size of the feeding structures should also be taken into consideration while designing arrays of these antenna as most of the valuable space is occupied by the feeding network portion. Different kinds of feeding array structures are proposed in various studies for the overall increase in the antenna performance either in terms of optimizing insertion loss or increasing the frequency bandwidth (e.g., SIW binary splitter, SIW power dividers, grounded coplanar waveguide (GCPW), T-junction power divider, combined T-type and Y-type dividers, four-way SIW power divider, a two-way power divider, and a 1-to-8 power divider network [42,43,44,45,46,47,48,49,50]), making power divider the most popular ones among all types of feeding network. However, most of the power dividers are in-phase power dividers limited to feed a certain number of antenna arrays providing the same amplitude and phase at the output which, when introduced at the system, provide the splitting of the beam at a higher frequency. In [51], the Vivaldi antenna array is fed by 1-to-4 non-uniform T-junction power dividers, limiting the number of antennas to four with a maximum measured gain of 11.3 dBi. In [47], a 1-to-8 power divider feed network is used for connecting to eight antipodal left and right arms for 5G mm wave application. Two distinct types of Vivaldi antenna for see-through-wall applications were developed in [52] for UWB using 8 × 1 and 16 × 1 Wilkinson power divider with a gain of more than 12 dBi and 13 dBi, respectively. What we see from the reported design of the power divider feeding network is that we can arrange 2^n^ number of antenna arrays for n number of rows of branching junction limiting the antenna to 2, 4, 8, 16... number of array sequence. Getting a 6, 10, 12... even antenna array combination sequence is impossible, and a lot of space is occupied by the feeding network within the array of antenna using this divider topology. A V-shaped even mode power divider with T- junctions is presented in [53] that limits the antenna array to four, constraining the maximum realized gain to approximately 11 dBi. Similarly, the microstrip feeding arrangement for this Vivaldi array antenna is in the same row, as seen in most of the antenna designs. Different prototypes of phase power dividers and impedance transformation are presented in the literature [54,55,56]. These bulky power dividers are mostly SMA connected, need multi-source signal feeding, and require power cables to connect to another system or antenna, which may lead to a loss in a system. Designing in-phase and out-of-phase power dividers within a limited area on a single substrate layer for direct feeding to antenna array system is still a challenging task. In addition, designing the antenna array system with the desired antenna combination and managing the feeding arrangement concerning the number of antennas in an array is still a tough problem. 

To overcome the limited arrangement of the antennas in an array, remove the constraint of adding feeding sources in a single row of Vivaldi antenna arrays, and eliminate the beam-splitting effect, and significantly enhance the radiation directivity of the antenna arrays, this paper presents a feeding network of a T-junction power splitter topology with the frequency-independent phase shifter added on one of the two arms. Each arm is then linked to three distinct sub-branches as a feeding source for the three Vivaldi antennas array. These three antennas at each arm are arranged compactly at multi-row locations separated by a length of guided wavelength so that the directivity is significantly increased, making the set up far more sensible for object detection within ultra-wideband region of (2.5–6.8 GHz) and (7.5–9.5 GHz). The U-shaped feed structures are placed in the arms, in between sub-branches, or the feeding source of the Vivaldi array, to compromise the signal delay to each feeding line. The antenna has a maximum realized gain of up to 14.12 dBi, with a front-to-back ratio of 23.23 dB. The high gain and directivity assure that the antenna can be used for microwave imaging of the hollow voids and objects buried under the concrete brick structure, as well as for applications requiring broad bandwidth communication. The paper is arranged as follows: Section 2 presents the design of the proposed antenna and its feeding structure, Section 3 consists of the parametric study of simulated and measurement results and discussion, Section 4 discusses the operation of the antenna with experimental results. Finally, Section 5 is the conclusion of the work.

## 2. Antenna Design

### 2.1. Antenna Structure

**Figure 1:**
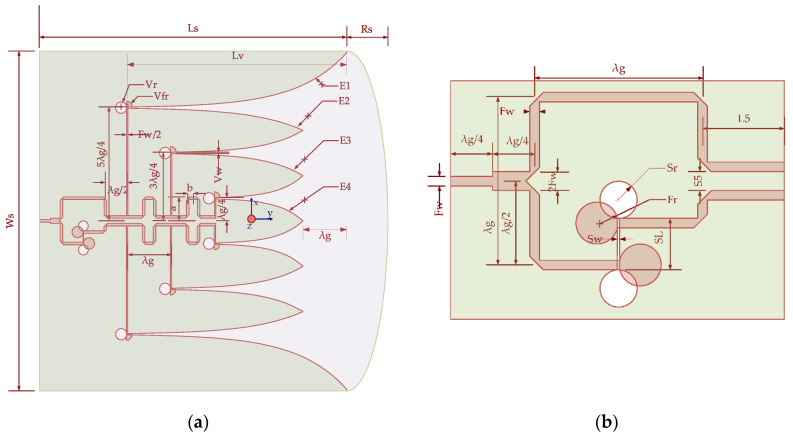
Structure of antenna: (**a**) top view of the antenna presenting feed, substrate, and ground layer structure; (**b**) enlargement of the feeding top view section of an antenna from Figure 1a.

The proposed geometry of the antenna is shown in Figure 1, where Figure 1a represents the top geometrical view of the substrate with the optimal dimension listed in Table 1, while Figure 1b shows the feeding portion of the antenna, which consists of a T-junction topology with an independent phase shifter at one of the arms. The antenna was designed on a Taconic substrate (ε_r_ = 4.3, tan δ = 0.0035). The size of the antenna is 167.48 mm × 158.25 mm × 0.6 mm. The antenna comprises a microstrip feeding network on the top side, and on the bottom side is a ground plane with an exponential tapering radiating patch. The 50-ohm quarter-wave microstrip line has a width “Fw”, which is followed by a T-junction base with a width twice that of the microstrip line where the junction base yields a symmetric T-junction power branch. The feed line length is set at λg/4, where λg is the guided wavelength of the center of the UWB frequency band. One of the power branches is routed through the frequency-independent phase shifter. This phase shifter changes the armed phase such that each power branch has 180+−20 -degree change in a phase shift which aids in the generation of a directive stable radiation pattern. Each arm is branched with three additional microstrip feed slots to the Vivaldi antenna array, with a width half the breadth of the arm. Each feed slot is separated from its constituent slot by a distance of ${\;\lambda }_{g}$ whereas its length varies by $\lambda_{\;g}/2$. The base of the Vivaldi slot antenna array is determined by the length and spacing of the feeding microstrip slot line. As shown in Figure 1a, the tapered slot, which is four exponential curves E1, E2, E3, and E4, is specified in terms of the values of the parameter as listed in Table 1, given by:

(1) $${\;E}_{1}:x=\frac{1}{2}(\;\frac{5\lambda_{g}}{2}+V_{w}(\exp\;(y\frac{\ln\left(\frac{\lambda_{g}}{2V_{w}}\right)}{L_{v}}\;)))\left(0\leq y\leq L_{v}\right),$$

(2) $${\;E}_{2}:x=\frac{1}{2}(\;\frac{5\lambda_{g}}{2}-V_{w}(\exp\;(y\frac{\ln\left(\frac{\lambda_{g}}{2V_{w}}\right)}{L_{v}-\lambda_{g}}\;)))\left(0\leq y\leq (L_{v}-\lambda_{g})\right),$$

(3) $$E_{3}:x=\frac{1}{2}(\;\frac{3\lambda_{g}}{2}-V_{w}(\exp\;(y\frac{\ln\left(\frac{\lambda_{g}}{2V_{w}}\right)}{L_{v}-2{*\lambda }_{g}}\;)))\left(\lambda_{g}\leq y\leq (L_{v}-\lambda_{g})\right),$$

(4) $$E_{3}:x=\frac{1}{2}(\;\frac{\lambda_{g}}{2}-V_{w}(\exp\;(y\frac{\ln\left(\frac{\lambda_{g}}{2V_{w}}\right)}{L_{v}-3{*\lambda }_{g}}\;)))\left(3\lambda_{g}\leq y\leq (L_{v}-\lambda_{g})\right),$$

### 2.2. Design of Feeding Structure

**Figure 2:**
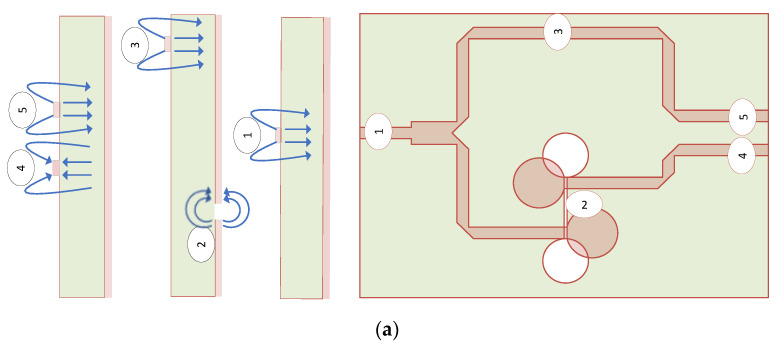

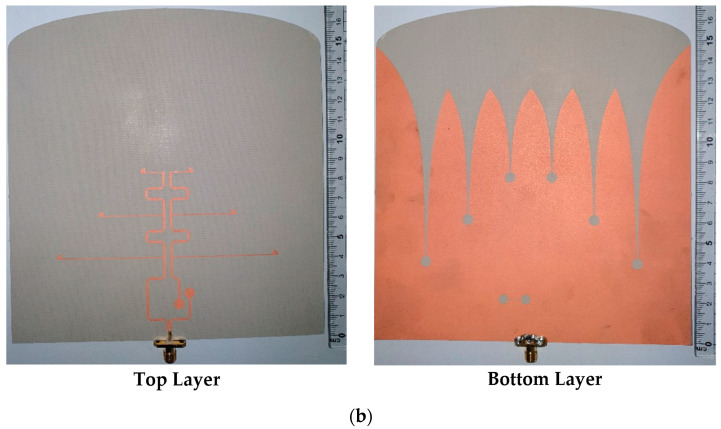
Proposed antenna with power divider: (**a**) power divider with E-field lines distribution at each segment 1, 2, 3, 4, 5 of a microstrip line; (**b**) top and bottom layer of the fabricated antenna.

The schematic of the proposed T-junction power divider topology feed by a frequency-independent phase shifter on one of the arms is shown in Figure 2a,b, representing the fabricated antenna prototype. The junction provides the slots with a signal uniform in magnitude and phase with characteristic slot line impedance of 50 Ohms. The phase shifter makes a 180+−20-degree phase shift on the microstrip line because of the microstrip to slot-line transition. As illustrated in Figure 2a, E-field lines at the output of segments 4 and 5 propagate in the opposite direction, which is integrated with the left and right arm of the proposed antenna. Both the arms are separated by a length twice the feed width, giving the common mode and differential mode impedance of 46.70 and 92.45 Ohms, respectively. 

**Figure 3:**
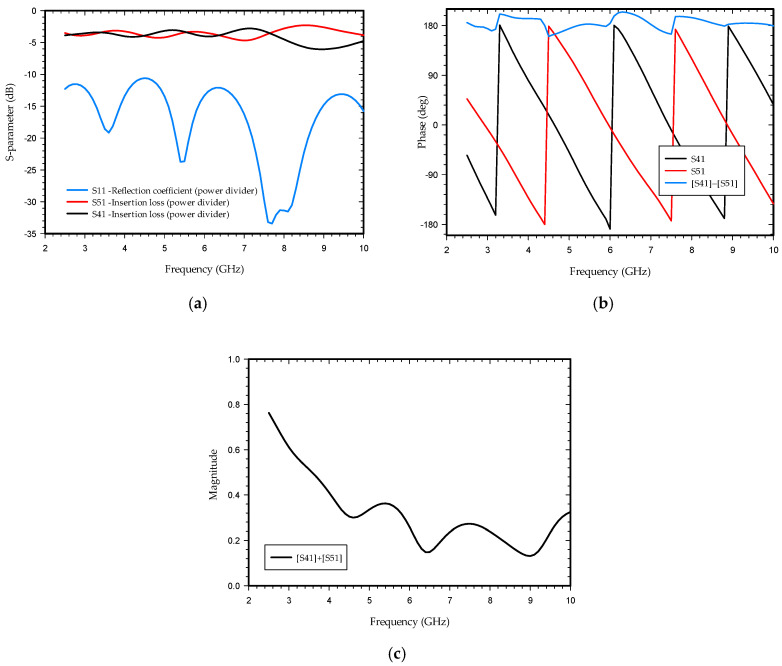
S-parameter with phase and magnitude of the power divider with phase-shifter at one of the arms: (**a**) output port return loss; (**b**) output port phase, and (**c**) magnitude.

As shown in Figure 3a, the simulating S-parameter of the power divider is below 10 dB, satisfying the operating frequency range of 2.5–10 GHz for the proposed antenna. The simulated insertion loss ranges from 2.1 dB to 5.7 dB. The power divider provides equal power divisions with a phase difference of 180 ± 20 degrees between the two output ports at segments 4 and 5 as observed in Figure 3b. Figure 3c shows the sum of the amplitude of the two segments of the power divider. The physical length of the arm of segment 4 differs because of fringing at the end of the circular stub and around the slot line (segment 2). This new effective length of the arm and power loss at microstrip slot line transition results in a change in phase and magnitude difference at the two ends of the segment. The change in beam tilt angle at the E-plane of the proposed antenna in the entire operating frequency is below nine degrees with a directive beam such that a small change in phase and magnitude of two feeding ports in this proposed antenna design is insignificant and does not hamper the directivity of the antenna. A considerable change in phase or magnitude difference may lead to beam split and a decrease in gain and directivity.

## 3. Results and Discussion

**Figure 4:**
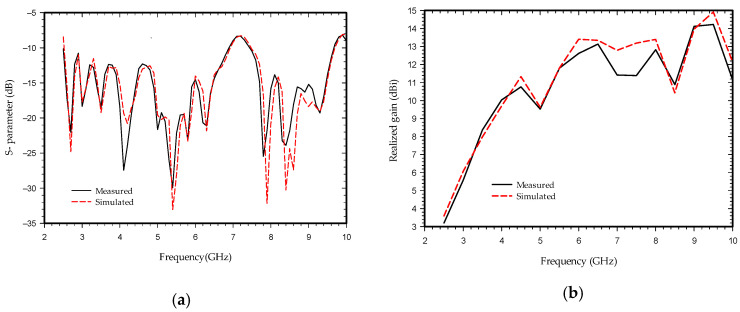
Simulated and measured results of the antenna: (**a**) return loss; (**b**) realized gain.

The commercially available high-frequency structure simulator (HFSS) software is used for the optimization and simulation of the proposed antenna. The simulation and measurement results are shown in Figure 4. Figure 4a shows impedance bandwidth of the proposed antenna below 10 dB from (2.5–6.8 GHz) and (7.5–9.5 GHz). The realized gain is shown in Figure 4b which is below 14.12 dB for the entire bandwidth with a close agreement between the measured and simulated results. The simulated and measured results vary slightly except at 4, 8, and 8.5 GHz, and can be endorsed by the losses taking place in the connector due to dimension imperfection and parasitic effect, imperfect soldering of the feed line with the connector, and fabrication errors during the etching process and characterization of the parameters of the substrate.

**Figure 5:**
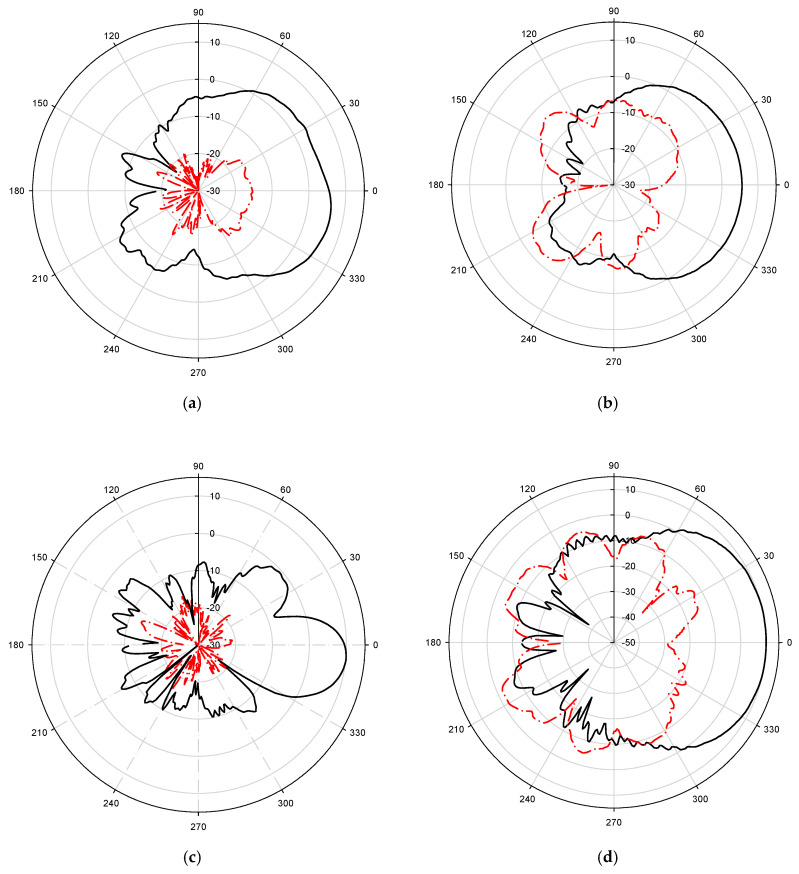

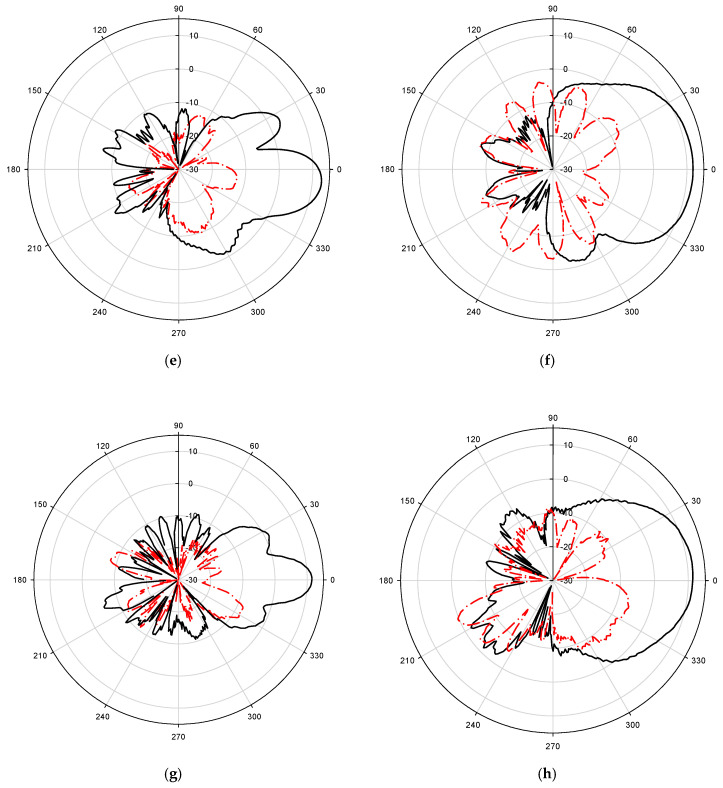

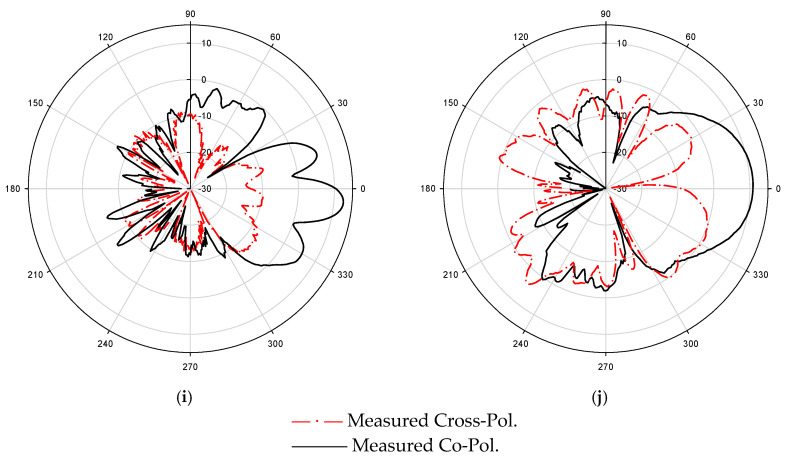
Measured far-field radiation pattern at E-plane (**a**,**c**,**e**,**g**,**i**) and H-plane (**b**,**d**,**f**,**h**,**j**) at frequency 3, 4, 5.5, 7, 8.5 GHz, respectively.

The measured 2D radiation patterns at 3, 4, 5.5, 7, and 8.5 GHz frequencies of the fabricated antenna are shown in Figure 5. The radiation patterns of the antenna with a frequency-independent phase shifter at one of the arms are almost directive in both the E-plane (x–y plane) and H-plane (z–y plane) which is one of the required characteristics for a Vivaldi antenna array.

**Figure 6:**
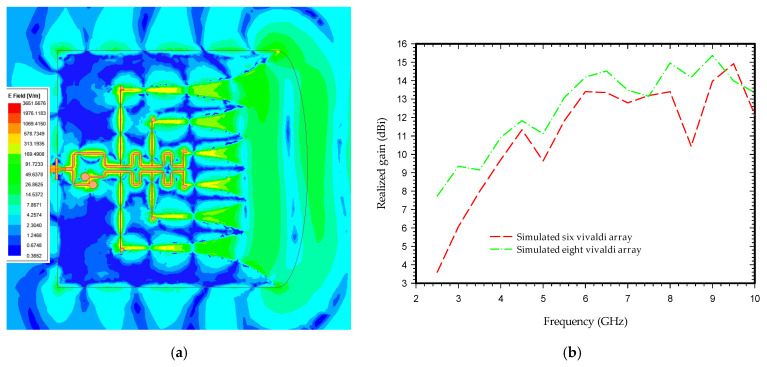

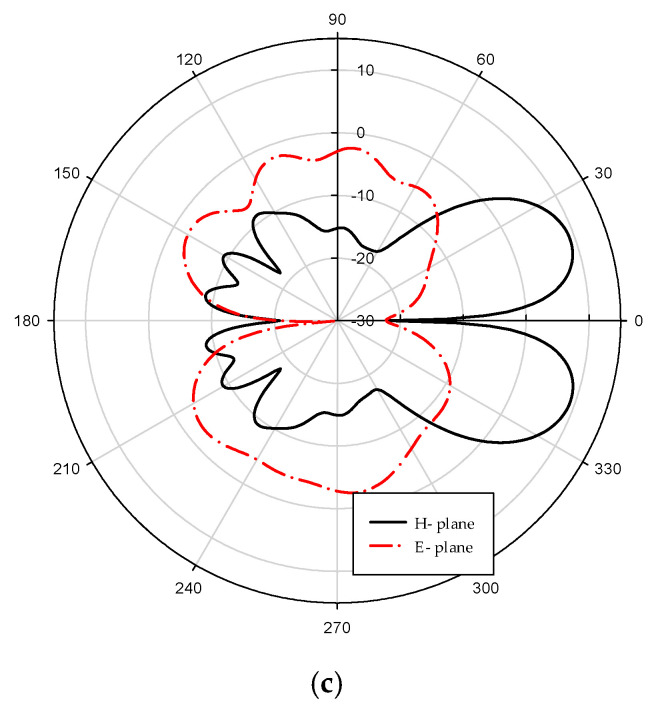
Simulated electric field, gain, and radiation pattern of the antenna: (**a**) electric field distribution at 4.5 GHz; (**b**) simulated realized gain of six and eight Vivaldi antennas array; (**c**) simulated radiation pattern at 4.5 GHz without phase shifter.

Figure 6a shows the simulated electric field distribution at 4.5 GHz frequency; it can be seen that the electric field radiated due to a change in surface current at each tapered slot of the Vivaldi array. These fields are superimposed to form the plane-like wave transmitted in the direction of wave propagation whose directionality is further enhanced by a semi-elliptical substrate of Length Rs. It can be seen from Figure 6b that the gain of the antenna rises with an increase in the number of antenna array segments achieving a maximum gain of up to 15.3 dBi. Figure 6c represents the simulated radiation pattern of the antenna when the frequency-independent phase shifter is replaced by a simple feed line. As shown in the radiation plot, the beam is split into two halves, losing the tendency to be directive, and has a reduced gain.

**Figure 7:**
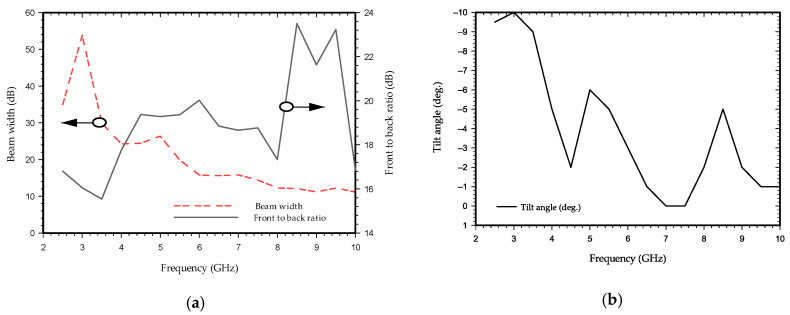
The measured variation of beam components with frequency: (**a**) front-to-back ratio and beam width; (**b**) E-plane beam tilts.

Figure 7a shows the front-to-back ratio and 3 dB beamwidth, while Figure 7b represents the beam tilt angle of the proposed antenna in the E-plane. The measured result of high front-to-back ratio at the operating bandwidth and minimum tilt angle of E-plane beam ensures that the antenna radiates maximum energy at the desired direction to enable penetration through the substrate, which is n for handling GPR and microwave imaging application.

The radiation performance and feed system of the suggested antenna is compared to that of previous known Vivaldi antennas array in Table 2. As indicated in the table, most antenna systems employ a T-junction power divider. In comparison to the existing ultra-wideband Vivaldi antennas array, the suggested antenna offers enhanced antenna array flexibility, feed management, and gain.

## 4. Experimental Study and Results

After validating the proposed antenna design, the experiment was performed in a room, in a controlled configuration measuring setup for microwave imaging. The objective of the test is to analyze and evaluate the change in the GPR images regarding the hidden targets placed within these hollow structures. These experiments are designed to enhance GPR survey techniques for quantifying the depth and width of hollow surfaces within a concrete block and locating running cables, pipes, or any metallic surface within it. 

### 4.1. Specimens 

**Figure 8:**
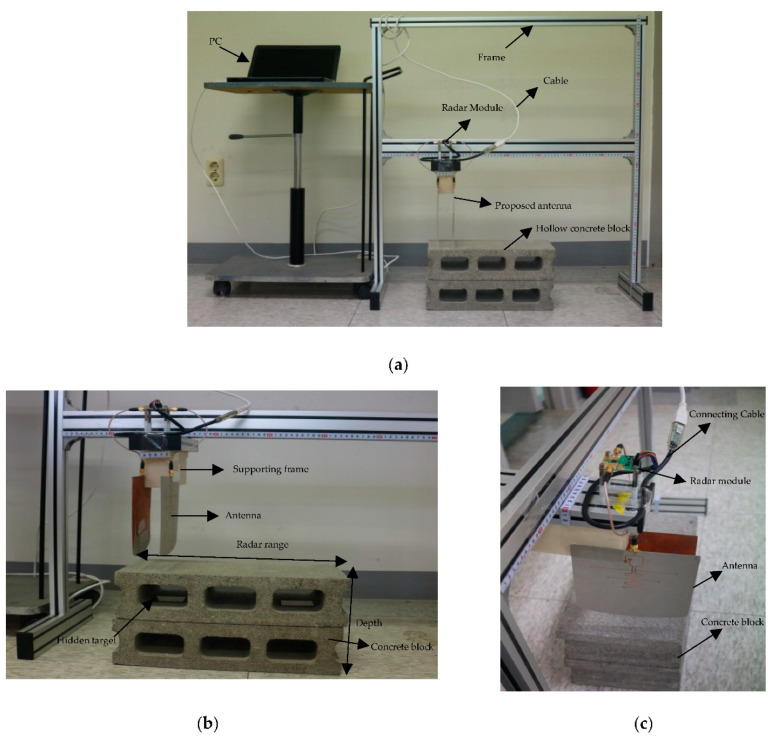
Experimental measurement setup: (**a**) setup arrangement with antenna scanning the concrete beam with the target within; (**b**) proposed antenna with radar module support and concrete brick and; (**c**) UWB radar module.

**Figure 9:**
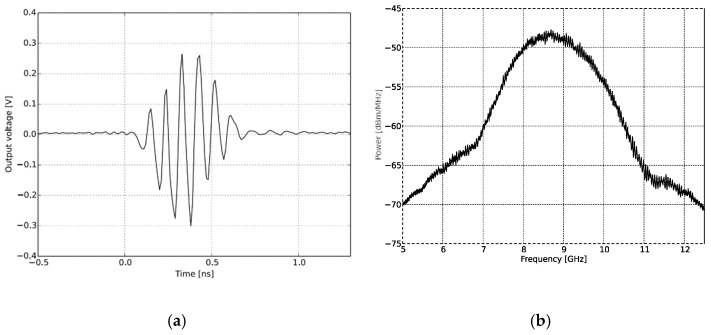
The transmitted pulse shape and frequency spectrum of IR-UWB radar for PGSelect = 10; (**a**) transmitted signal in the time domain and; (**b**) transmitted signal pulse in the frequency domain.

The setup consists of a UWB radar module (NVA-R661 of Xethru Co., Oslo, Norway), RF cables, supporting frame, and connectors connecting to PC and antenna modules as shown in Figure 8a. The specimens represent an aerated concrete brick and hidden substrate plates. The concrete block is 38.5 cm × 18.5 cm × 10 cm in size, with three hollow-spaced structures measuring 9 cm × 5 cm each 2.5 cm apart are shown in Figure 8b. A substrate plate with dimensions of 5.5 cm × 4.5 cm × 0.18 cm was taken as a target. The X2 chip of the NVA-R661, as shown in the Figure 8c module, generates and transmits UWB pulses of high-order Gaussian impulse signal with several GHz bandwidths of signal duration in the order of nanoseconds [58]. The high-frequency signal was chosen based on the maximum gain and return loss of the antenna, as well as the radar ability to have high-resolution depth throughout the object. The impulse signal was set to around the operating bandwidth of the antenna by selecting number 10 of the PGSelect command, which has a center frequency ($f_{\;c}$) at 8.8 GHz and 3.1 GHz bandwidth with peak-to-peak output amplitude of 0.54 volts. This satisfied the minimum separation of antenna distance, d = 5 cm facing parallel to each other by the condition of $d>\lambda_{\;c}/4$. The radar resolution per frame depth was set to 0.20 cm on air, and when accounting for the concrete brick of relative permittivity ε_r_ = 2.5, at 8.8 GHz, a resolution depth of 0.16 cm was considered. Matlab is used for signal analyzing and processing. Figure 9 shows the time and frequency domain response of transmitted signal pulse in order of nanoseconds for the selected ($f_{\;c}$) at 8.8 GHz.

### 4.2. Signal Analyzing

**Figure 10:**
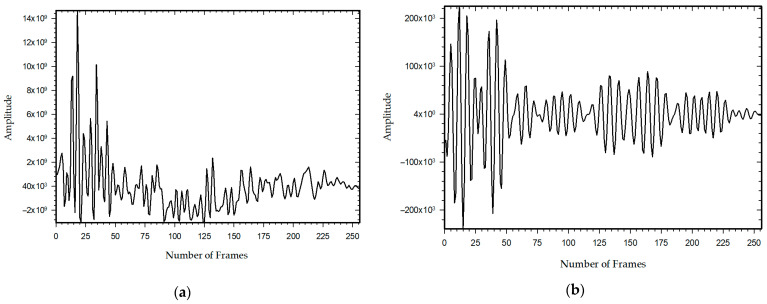
The received pulse shape and correlated signals from IR-UWB radar for PGSelect = 10; (**a**) received raw signal strength and; (**b**) correlated signal pulse.

The reflected signal from the target consists of a clutter signal, target signal, and noise is combinedly represented as:

ri = rc,i + rt,i + rn,(5)

where ri is the i-th received signal, consisting of clutter signal rc,i, the target signal rt,i, and the noise rn. The received raw signal, as shown in Figure 10a, is then cross-correlated with the template signal (Figure 9a). These template signals are the transmitted signal generated in the X2 chipset. This helps the signal to achieve the best enhancement in the signal-to-noise ratio and correlated the pattern of the received signal concerning target position. A correlated signal is shown in Figure 10b. The n-th correlated signals are taken and grouped covering the whole specimen as a single bin and then the Fourier transform is applied to those signals. The Welch’s power spectral density is estimated from the Fourier transform targeted signals and finally, holographical slices of 2-D space image are obtained detecting the position of the hidden target.

### 4.3. Measurement Setup

**Figure 11:**
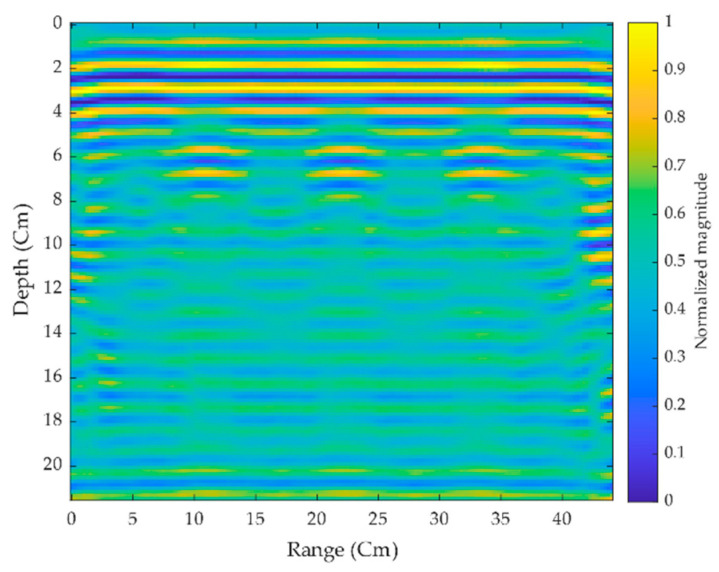
Scanned raw data from the radar module.

The experiment is carried out with three hollow-shaped aerated concrete brick structures stacked on top of one another, with some metallic objects inserted within. The main objective is to locate the concealed targets within these hollow structures. The proposed antenna was placed 2.5 cm above the concrete structure’s surface, and scans were performed manually with an interval of 0.25 cm per step. By summing the reflected echo signals for each antenna position, each scan progressively forms an image. The echo thus obtained by each antenna position is considered as a pixel that is shifted to match or align with the next scan pixel elements in the image map. A total of 176 scans were conducted to generate a complete 2-D image of the scanned surface, as shown in Figure 11, where the figure represents the raw scanned data taken from the radar module before further applying signal processing. A substrate plate is placed as a target within the hollow-shaped concrete brick at the depth of 7.5 cm from the surface. The experiment is made in three scenarios.

**Figure 12:**
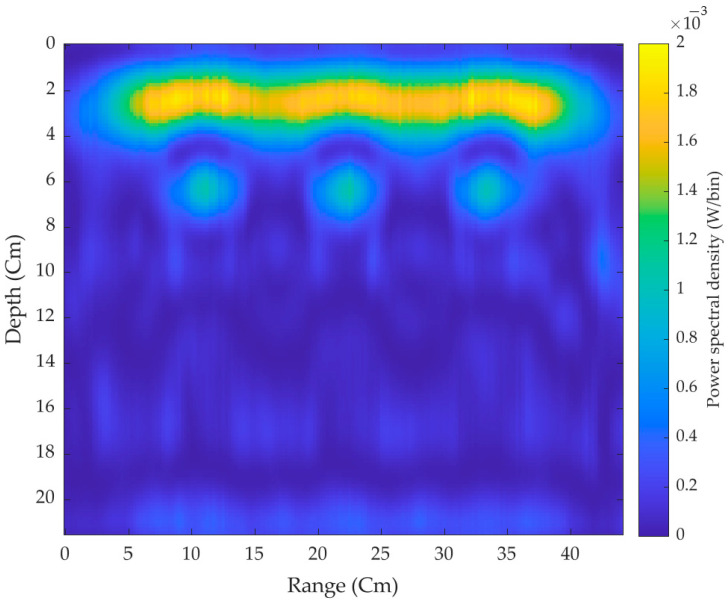
2-D cross-surface scanned image without the target.

First, the pair of antennas made the scans throughout the concrete brick without placing the target plates. Figure 12 shows the power spectral density of the three hollow 2-D cross surfaces of the brick without targets where it can be seen that the signals are reflected strongly from the air-brick surface. On moving further down through the section of the brick, the three hollow surfaces can be easily seen with significant penetration depth. 

**Figure 13:**
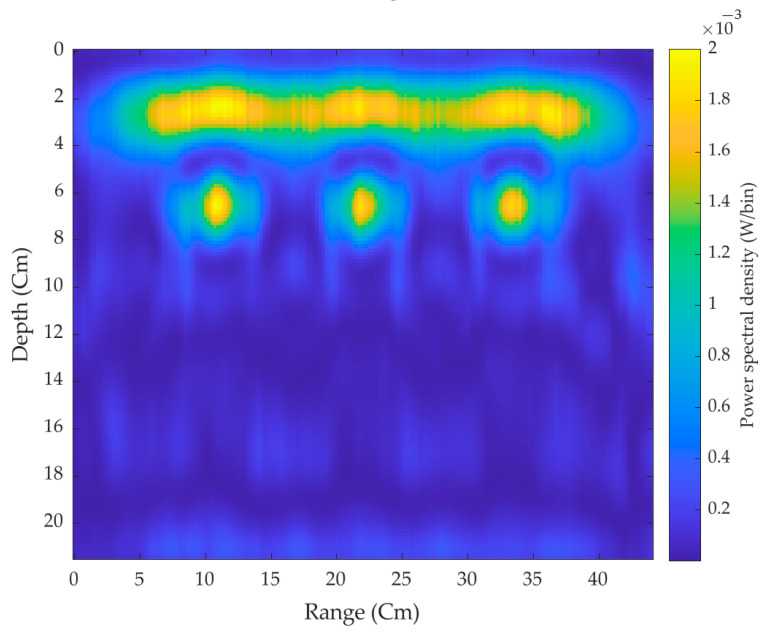
2-D cross-surface scanned image with three targets.

Second, all the three target plates are placed inside the hollow concrete brick, and scans were made across the surface. As shown in Figure 13, signals are massively reflected below the first layer of the air-surface interference indicating the presence of three hidden targets.

**Figure 14:**
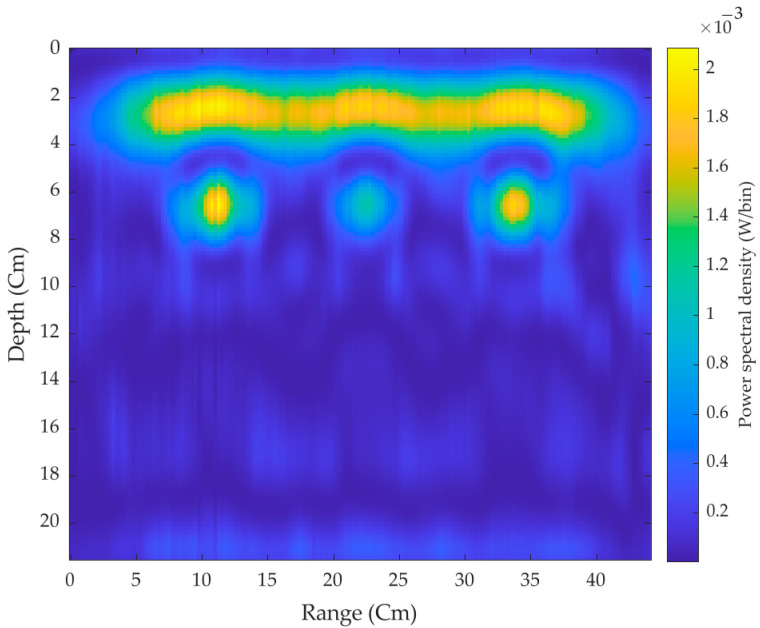
2-D cross-surface scanned image with one target removed.

After this, one middle target is removed from the hollow concrete brick, and scans are again performed where the remaining two targets can be seen as shown in Figure 14. From all those three cases, looking at the scanned image we can easily predict the hollow surfaces and the hidden targets at the position around 7.5 cm from the antenna. The slight discrepancy between the real and the predicted target position of the scanned image is due to the variation in permittivity of hollow concrete brick. Poor reflectivity and strong attenuation in the hollow interior of the brick hinder further tracking of in-depth microwave imaging. The main advantage of this lightweight antenna is that it facilitates a higher gain and narrow beam width over a wider frequency range, suitable for air-coupled GPR. The limitation of the proposed antenna is that it cannot be utilized as a ground-coupled antenna because of the orientation of radiated beam and antenna shape, making it unable to obtain clearer data and greater depth of inspection for small surface anomalies such as cracks.

## 5. Conclusions

A novel feed system consisting of high gain six-slot Vivaldi antenna arrays on a single substrate layer using a power splitter based on binary T-junction power splitter topology and frequency-independent phase shifter has been presented. The antenna exhibits an out-of-phase feeding mechanism in combination with proper Vivaldi antenna array layout on separate rows causing high realized gain and a front-to-back ratio up to 14.12 dBi and 23.23 dB, respectively, within ultra-wideband regions of (2.5–6.8 GHz) and (7.5–9.5 GHz). Broad bandwidth, high gain, and strong directivity are all benefits of the suggested antenna, making it a viable option for applications that need broad bandwidth communication. The feeding system overcomes the limited arrangement of the antenna in an array reported in the literature, removes the constraint of adding feeding sources of Vivaldi antenna arrays in a single row, eliminates the beam-splitting effect, and significantly enhances the radiation directivity of the antenna arrays. The fabricated antenna is deployed for the detection of substrate plates as targets placed inside the concrete brick. To begin, scans were taken on the concrete brick surface. The power spectral density of the scanned image reviled the three hollow spaces within the brick. Second, all three target plates were placed into a hollow concrete brick revealing the scanned image with three targets, and finally, one of the targets was removed and scans were conducted, revealing the scanned image without one of the targets. The 2-D imaging of the systems detecting the target location and a hollow space inside the concrete brick confirms that the proposed antenna is suitable for use in microwave imaging and GPR application.

## Figures and Tables

**Table 1:** Parametric dimensions of the proposed antenna structures.

Parameter	mm	Parameter	mm
Fw	1.2	Sw	0.3
λg	21.1	Vw	0.3
Rs	0.125 × Ws	Sl	λg/2
Sr	λg/8	S5	2 × Fw
Fr	λg/8	L5	10.25
Vr	λg/8	Ls	147.7
Vfr	λg/8	Ws	158.25
Lv	4 × λg	a+b	λg/4

**Table 2:** Comparison table.

Ref.	Number of Vivaldi Antenna Elements	Frequency Range (GHz)	Feed System	Gain (dBi)
[45]	8	9–11	T-junction power divider and GCPW transition	12
[47]	8	24.75–28.5	T-junction power divider	6.96–11.32
[51]	4	1–6	T-junction power divider	5.7–11.3
[53]	4	2–11	V-shaped power divider with T-junction	3.75–11
[57]	4	25–31	4 × 4 butler matrix	10.2
Proposed antenna	6	2.5–6.8 and 7.5–9.5	T-junction power divider with independent phase shifter	3.2–14.12

## Data Availability

Data sharing not applicable.
